# Identification of dental implants using deep learning—pilot study

**DOI:** 10.1186/s40729-020-00250-6

**Published:** 2020-09-22

**Authors:** Toshihito Takahashi, Kazunori Nozaki, Tomoya Gonda, Tomoaki Mameno, Masahiro Wada, Kazunori Ikebe

**Affiliations:** 1 Department of Prosthodontics, Gerodontology and Oral Rehabilitation, Osaka University Graduate School of Dentistry, 1-8 Yamadaoka, Suita, Osaka, 565-0871 Japan; 2 Division of Medical Information, Osaka University Dental Hospital, 1-8 Yamadaoka, Suita, Osaka, 565-0871 Japan

**Keywords:** Deep learning, Dental implant, Artificial intelligence, Object detection, Yolov3

## Abstract

**Background:**

In some cases, a dentist cannot solve the difficulties a patient has with an implant because the implant system is unknown. Therefore, there is a need for a system for identifying the implant system of a patient from limited data that does not depend on the dentist’s knowledge and experience. The purpose of this study was to identify dental implant systems using a deep learning method.

**Methods:**

A dataset of 1282 panoramic radiograph images with implants were used for deep learning. An object detection algorithm (Yolov3) was used to identify the six implant systems by three manufactures. To implement the algorithm, TensorFlow and Keras deep-learning libraries were used. After training was complete, the true positive (TP) ratio and average precision (AP) of each implant system as well as the mean AP (mAP), and mean intersection over union (mIoU) were calculated to evaluate the performance of the model.

**Results:**

The number of each implant system varied from 240 to 1919. The TP ratio and AP of each implant system varied from 0.50 to 0.82 and from 0.51 to 0.85, respectively. The mAP and mIoU of this model were 0.71 and 0.72, respectively.

**Conclusions:**

The results of this study suggest that implants can be identified from panoramic radiographic images using deep learning-based object detection. This identification system could help dentists as well as patients suffering from implant problems. However, more images of other implant systems will be necessary to increase the learning performance to apply this system in clinical practice.

## Background

Dental implants were developed in the 1980s [1], and they are now used for patients with missing teeth globally. Their effect on dental treatment is great, and various improvements in patients’ quality of life have been reported [2, 3]. Implant treatment is no longer unusual for either patients or dentists. However, because more than 30 years have passed since implants were introduced into clinical practice, various implant problems have been also reported, such as complications in the superstructures or implants [4] and peri-implantitis [5]. Additional prosthodontic, periodontic, or surgical treatments are needed to solve these problems. When performing these treatments, various information is needed about the intra-oral implant, such as the manufacturer, system, length, and width of implant, method of fixation, and type of abutment. If the implant patient was previously treated at the same family clinic, this information is easy to obtain from the patient’s medical record. However, if the treatment was performed at another clinic and the patient cannot contact the treatment provider, this information may be difficult or impossible to obtain. Recently, some patients with implant troubles have visited other clinics for various reasons, such as relocation or the closure of family clinics. In such cases, dentists must identify the patient’s implant information from the limited data obtained from oral photographs, radiographs, a study model, and so on. In particular, the type of implant system must be identified in order to conduct additional treatments. Dentists with sufficient knowledge about and experience of implant treatments can identify implant systems and perform treatment, but those without the knowledge and experience cannot identify the system and treat the patient. Therefore, there is a need for a system for identifying the implant system of a patient from limited data that does not depend on the dentist’s knowledge and experience.

Artificial intelligence (AI) technology has been applied in various fields, and its presence is already essential in many of them. In AI technology, there are several methods that are used in accordance with the task. In medicine, AI has already been used for robotics, medical diagnosis, statistics, and human biology-up [6]. A deep learning method, one of the AI technologies, is adequate for prediction, object detection, classification, and other similar tasks. In dentistry, the diagnosis of dental diseases using oral or X-ray images [7], prediction of treatments [8], classification [9], statistics from research data [10], and other topics have been addressed using a deep learning method. Specifically, studies on the diagnosis of diseases using a deep learning have increased, and deep learning-based object detection algorithms for images are usually used for this task [11]. The ability of diagnostic systems using deep learning is already comparable or superior to that of humans, and these systems will help prevent dentists from missing problems or making errors. If this system also can be applied for identifying implant systems using dental X-ray images, it will help both dentists and patients solve implant problems.

The purpose of this study is to develop an automated system for identifying implant systems using a deep learning-based object detection method. The hypothesis of this study was that this system could detect and identify the implant.

## Methods

### Data collection

Panoramic radiographs were obtained from patients who received implant treatment in the Department of Prosthodontics, Gerodontology and Oral Rehabilitation at Osaka University Dental Hospital after January 2000. Panoramic radiographs with unknown implants were excluded and totally 1282 images were used to annotate implants. All images were JPEG files that were resized to 416 × 416 pixels. The images were randomly divided into two datasets: one for training (1026 images, 80%) and one for testing (256 images, 20%). Training datasets were used to make the model by learning, and the testing dataset was independent of the training dataset and used to assess the performance of models which was made using a training dataset.

### Annotation of implants

Six implant systems manufactured by three companies were annotated manually in all panoramic radiographs using an annotation tool (labelImg). They consisted of four systems, which have straight apex: MK III and MK III Groovy (MK III/IIIG) by Nobel Biocare (Zürich, Switzerland), bone level implant (BL) by Straumann (Basel, Switzerland), and Genesio Plus ST (Genesio) by GC (Tokyo, Japan); and two systems which have tapered apex: MK IV and Speedy Groovy (MK IV/SG) by Nobel Biocare.

### Deep learning algorithm

To implement the object detection algorithm, Python 3.5.2 and the Keras library 2.2.4 were used with TensorFlow 1.12.0 as the backend. The object detection application, You Only Look Once (YOLO) v3 [12], with fine-tuning was used, and the dataset was trained to detect implants. The training dataset was separated into 16 batches for every epoch, and 1000 epochs were run with a learning rate of 0.01.

### Assessment of the learning result

**Fig. 1:**
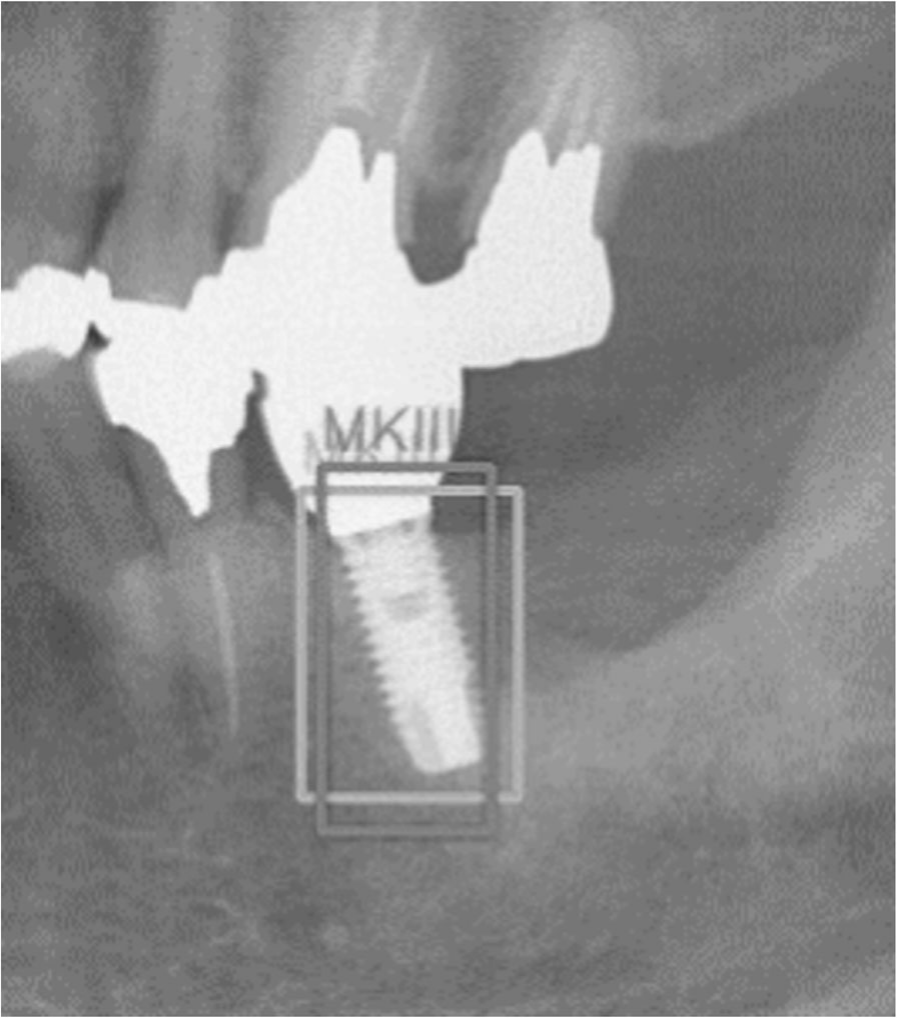
Sample image for calculating IoU (MK III implant). The light gray square indicates the ground-truth bounding box, and the dark gray square indicates the predicted bounding box. IoU value was calculated that the overlapped area of light gray and dark gray squares was divided by the united area of light gray and dark gray squares

The total number of the implant system in all panoramic radiographs, number of implant system identified correctly (true positives; TP), and those identified as other types of implant system (false positives; FP) were identified. The average precisions (AP) of each implant system, the mean average precision (mAP) of an intersection over unit (IoU) of more than 0.5, and mean IoU (mIoU) were calculated. IoU was calculated as follows (Fig. 1).

IoU = area of overlap (both ground-truth bounding box and predicted bounding box)/area of union (either ground-truth bounding box or predicted bounding box)

APs are higher in value depending on the IoU threshold. In this study, the IoU threshold was determined to be 0.5, which is the value commonly used in other studies on object detection [13]. In addition, mAP is calculated by taking the average of AP over all classes. Higher values indicate that the learning is more accurate.

## Results

**Fig. 2:**
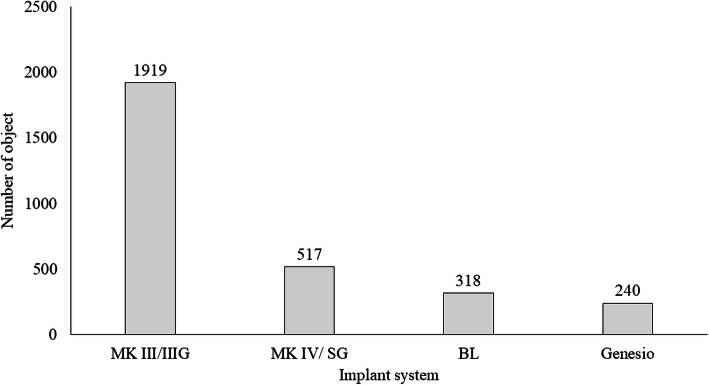
Total number of objects of each implant systemin all images. MK III/MK III Groovy: MK III/IIIG, MK IV/Speedy Groovy: MK IV/SG, bone level: BL and Genesio Plus ST: Genesio

**Fig. 3:**
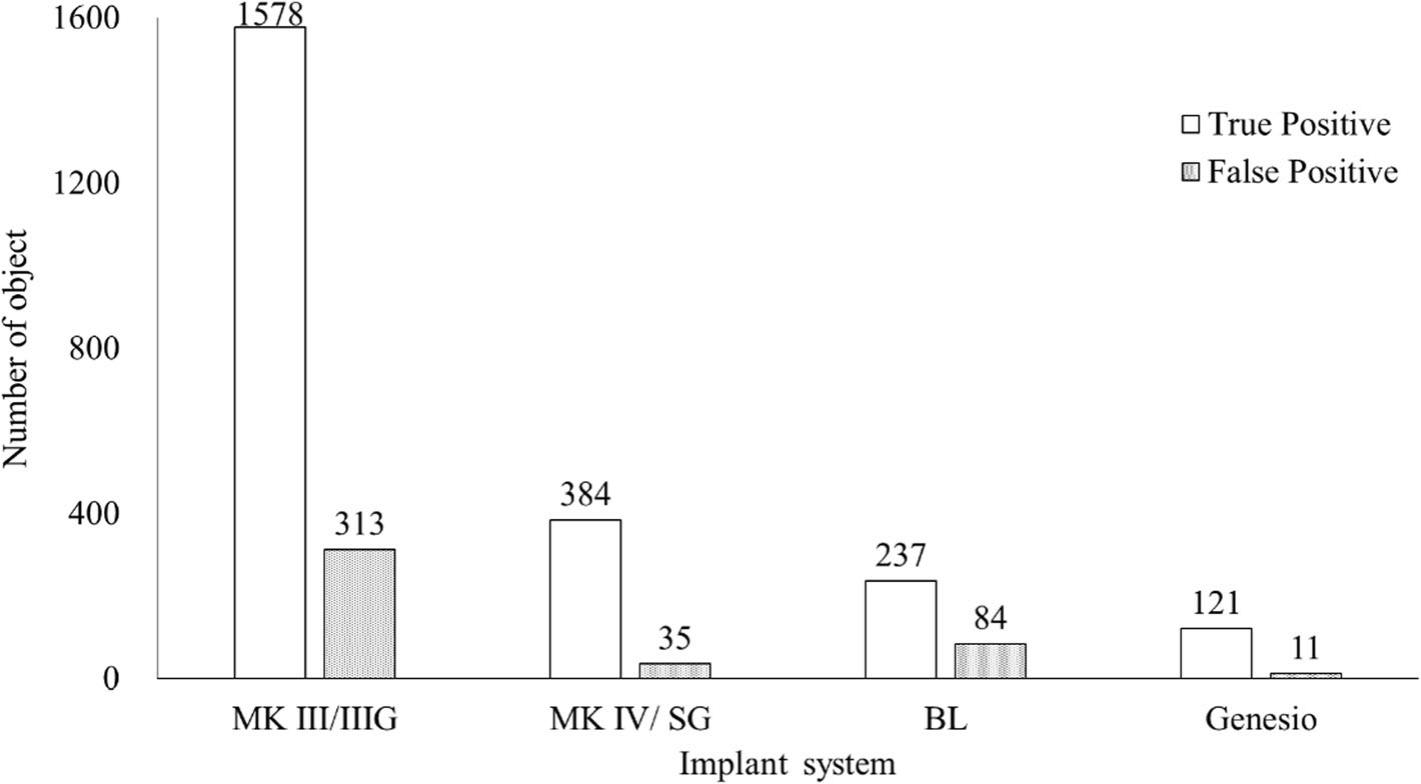
The total number of implant systems detected correctly (TPs) and those detected as other prostheses (FPs). MK III/MK III Groovy: MK III/IIIG, MK IV/Speedy Groovy: MK IV/SG, bone level: BL and Genesio Plus ST: Genesio

**Fig. 4:**
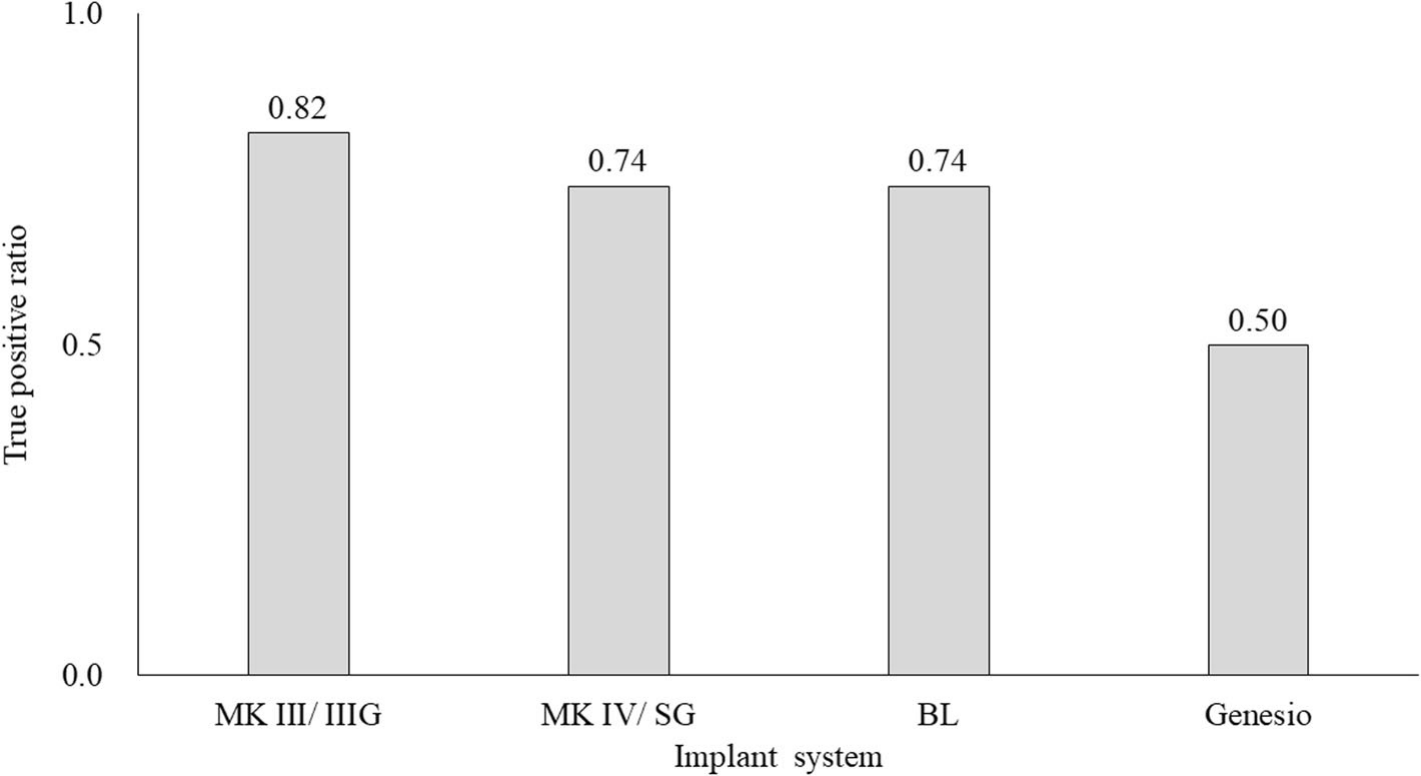
Ratio of implant systems detected correctly to all detected systems (True Positive ratio). MK III/MK III Groovy: MK III/IIIG, MK IV/Speedy Groovy: MK IV/SG, bone level: BL and Genesio Plus ST: Genesio

**Fig. 5:**
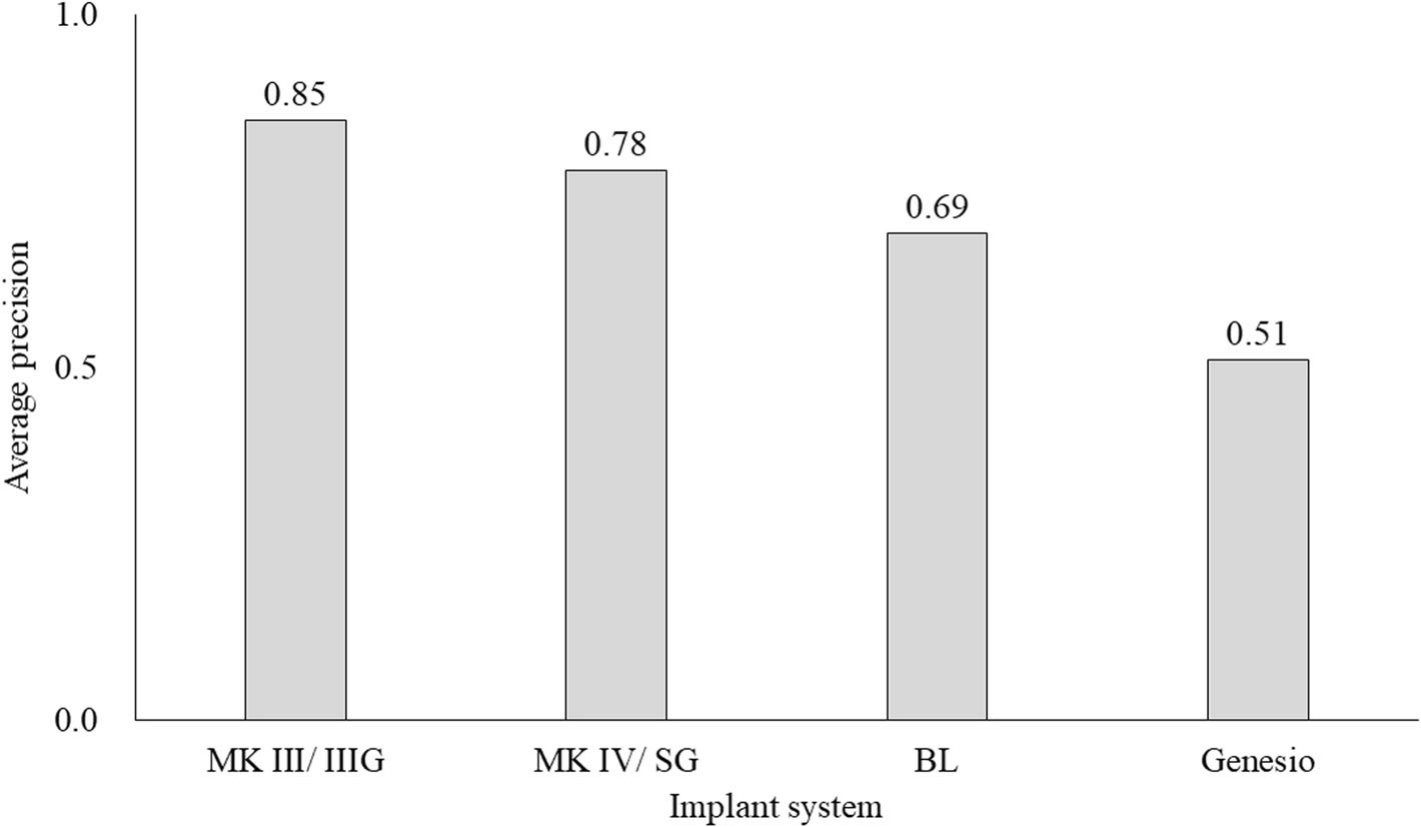
Average precision (AP) of each implant system in all images. MK III/MK III Groovy: MK III/IIIG, MK IV/Speedy Groovy: MK IV/SG, bone level: BL and Genesio Plus ST: Genesio

At least 240 instances of each implant system were detected in the panoramic radiographic images: the most common type was MK III/IIIG (1919 instances) and the least common was Genesio (240 instances; Fig. 2). The number of implants detected correctly (True Positive: TP), and those detected as other systems (false positive: FP) are shown in Fig. 3. The number of both TP and FP were the largest in MK III /IIIG and the smallest in Genesio. The TP ratios ranged from 0.50 to 0.82; the highest value was obtained for MK III/IIIG, and the lowest was obtained for Genesio. The values of MK IV/SG and BL were the same (Fig. 4). In Genesio, half of them could not be detected correctly. The APs of each implant were as follows: MK III/IIIG: 0.85, MK IV/SG: 0.78, BL: 0.69, and Genesio: 0.51 (Fig. 5). MK III/IIIG and MK IV/SG could be detected with high accuracy. The mAP and mIoU of this identification system were 0.71 and 0.72, respectively.

## Discussion

There are some problems with implants that cannot be solved in general clinics. In these problems, an unknown implant system will make the problems worse. Therefore, the identification of an implant system is necessary for both dentists and patients, and an automated identification system that is not dependent on the dentist’s expertise is needed. Considering these issues, an AI-based approach seems to be a potentially suitable solution, and this study was conducted to focus on developing an automated identification system of implants from panoramic radiographic images using object detection. There are already two methods for implant identification [14, 15]. In the first, dental radiographic images of many implant systems have been uploaded to a website and dentists are able to check them to find an image that matches the patient’s implant image. The second system employs nine questions about implant characteristics. The database returns candidate matching implants based on the answers to these questions, and dentists must match them with those of the patient. Both of these systems require dentists to check whether two images of an implant are the same to identify the implant system. In contrast, the system in this study is based on deep learning, one of AI techniques, and not a dentist but the computer itself identifies the implant.

When evaluating the performance of the object detection, two indices, mean average precision (mAP) and mean intersection over union (mIoU), were mainly used. mAP is used to measure the accuracy of object detection model, and the closer the value is to 1.0, the more accurate the model is. A mAP of more than 0.7 seemed to be regarded as a good value in other studies [13], but there is no clear criterion. A mIoU of more than 0.7 is regarded as a good value [16, 17], and the mAP the mIoU obtained in this study are 0.71 and 0.72, respectively. Considering these, the performance of this learning system can be considered to be high. The values of the hyperparameters were determined from the results of preliminary experiments with various combinations of values. Learning with this combination yielded superior mIoU and mAP values than other combinations.

In the results of this study, the AP of each implant system varies from 0.51 for Genesio to 0.85 for MK III/IIIG, and the mAP is 0.71. The TP ratios also vary from 0.50 for Genesio to 0.82 for MK III/IIIG. These differences are caused by the number of implants, their locations, and their similarity of shape. When selecting implant systems to recognize in this study, frequently used implant systems were selected because the number of implants seemed to be one of the most important factors. In fact, both AP and TP ratio of Genesio, which was the least number of images, were the minimum value, and those of MK III/IIIG were the maximum. About 1300 panoramic images and a total of 3000 implant images were used, but these numbers were not enough to recognize all the implant systems included. To increase the learning performance, a sufficient number of implant images are necessary.

**Fig. 6:**
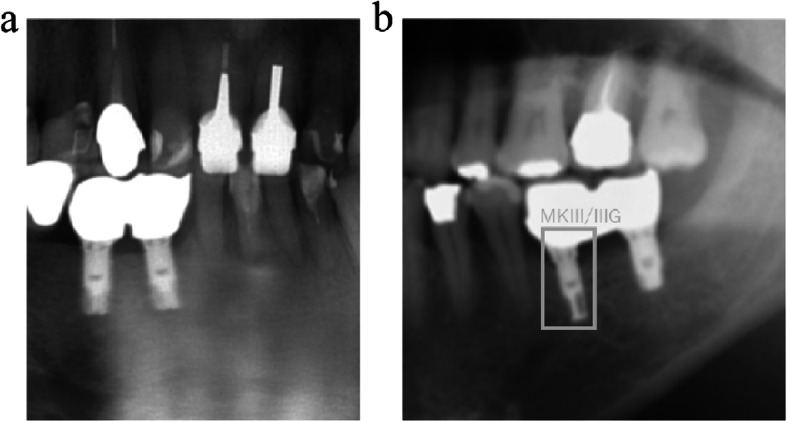
Sample images of misdetected implants. **a** Both implants could not be detected because of the shadow of the spina. **b** Left implant was detected correctly as MK III/IIIG, but the right implant was not detected because of an unclear image

To identify implant systems from radiographic images, dental radiography, panoramic radiography, and computed tomography were considered. In this system, it is thought that implant systems are identified by the shape of the collar, groove, and apex of the implant images, which are unique characteristics of each implant. Consequently, the quality of the training images is important so that these shapes of the implants can be recognized in detail. The advantage of using panoramic radiographic images is that they are standardized to a certain level regardless of the patient, and the shapes of the implants in the images are also standardized. However, the disadvantage is that the implant shapes are unclear when they overlap with a shadow, such as the spina or floor of the maxillary sinus, or when they were too short or much inclined. This may cause misdetection, and some misdetections actually occurred in the result of this study (Fig. 6). In such cases, the images of dental radiography may be more useful. Another disadvantage is the shape of the images. The shape of the images in the learning procedure of this algorithm is square, but the original panoramic radiographic images are rectangular. Therefore, in the learning procedure, panoramic radiographic images are laterally compressed and the shapes of the implants are also compressed. As a result, implant details become unclear, and this could decrease learning performance. The learning performance could be increased by cropping the original panoramic image into a square shape that includes implants beforehand.

In this study, four systems by one manufacturer and more two systems by two manufactures were selected. The reason was to know how much of a difference this system could identify. In the results, the misidentification between MK III/IIIG and Genesio often occurred, especially some of Genesio were misidentified as MK III/IIIG. They are all straight type, and the differences among them are subtle: differences among three systems are the shape of the platform and apex. These small differences are not easy to distinguish in compressed images and misidentification hence occurred. Increasing images with high quality must also be a solution to prevent these misidentifications. In addition to the shape of apex or collar, other differences, such as the shape of the inner screw or space between the bottom of the inner screw and implant body, may be helpful to identify similar-shaped implant.

## Conclusion

Though there are several issues that still need to be addressed, implant systems can be identified from panoramic radiographic images using deep learning-based object detection. To increase the learning performance and apply this system in clinical practice, a higher quality and larger number of implant images and images of other implants will be needed in subsequent studies.

## Data Availability

The datasets generated and/or analyzed during the current study are not publicly available because the panoramic radiographs used in this study can be used only in the hospital.
